# Adaptive Trust Threshold Model Based on Reinforcement Learning in Cooperative Spectrum Sensing

**DOI:** 10.3390/s23104751

**Published:** 2023-05-14

**Authors:** Gang Xie, Xincheng Zhou, Jinchun Gao

**Affiliations:** 1 School of Information and Communication Engineering, Beijing University of Posts and Telecommunications, Beijing 100876, China; 2 School of Electronic Engineering, Beijing University of Posts and Telecommunications, Beijing 100876, China; zhouxc@bupt.edu.cn; 3 Beijing Key Laboratory of Work Safety Intelligent Monitoring, Beijing University of Posts and Telecommunications, Beijing 100876, China; gjc@bupt.edu.cn

**Keywords:** cooperative spectrum sensing, SSDF, intelligent malicious user, trust model, Q-learning

## Abstract

In cognitive radio systems, cooperative spectrum sensing (CSS) can effectively improve the sensing performance of the system. At the same time, it also provides opportunities for malicious users (MUs) to launch spectrum-sensing data falsification (SSDF) attacks. This paper proposes an adaptive trust threshold model based on a reinforcement learning (ATTR) algorithm for ordinary SSDF attacks and intelligent SSDF attacks. By learning the attack strategies of different malicious users, different trust thresholds are set for honest and malicious users collaborating within a network. The simulation results show that our ATTR algorithm can filter out a set of trusted users, eliminate the influence of malicious users, and improve the detection performance of the system.

## 1. Introduction

With the rapid development of wireless communication technology, the demand for wireless communication equipment and Internet of Things equipment has expanded rapidly, and the available spectrum resources are becoming increasingly scarce. However, due to the existing static spectrum allocation scheme, most of the licensed radio spectrum is not efficiently utilized. According to a research report by the Federal Communications Commission (FCC) of the United States, licensed spectrum utilization ranges from 15% to 85% [1]. In order to solve the contradiction between the shortage of spectrum resources and the low utilization of the spectrum, Dr. J. Mitola et al. have proposed the concept of the cognitive radio network (CRN) [2], which allows a secondary user (SU) with sensing ability to sense and use the spectrum of a nearby primary user (PU) without interfering with the communication of the primary user [3]. Spectrum sensing is a necessary prerequisite in cognitive radio systems. Cooperative spectrum sensing (CSS) can avoid the impact of shadowing and multipath fading, make full use of spatial differences, and overcome the shortcomings of single-user spectrum sensing [4,5]. However, malicious users (MUs) in the network may send incorrect data when uploading data to the fusion center (FC) to achieve their own purposes. This phenomenon is called a spectrum-sensing data falsification (SSDF) attack [6,7].

An ordinary SSDF attack is also known as a “Byzantine attack” [8], and its attack methods mainly include the following: “always yes”, “always no”, “always false”, and “Ffixed probability”. In order to defend against ordinary SSDF attacks, some scholars have conducted corresponding research [9,10,11,12,13,14,15,16,17,18,19,20,21]. In [12], a method based on Bayesian detection was used to defend against SSDF attacks, but it needs to detect a fixed number of nodes, which consumes a large amount of energy on the system. Lu et al. explained that when a system is attacked by SSDF independently or jointly launched by MUs [13], it can defend against malicious users (MUs) with the help of trusted SUs, that is, by only relying on the data of trusted SUs during data fusion, it can find MUs. This algorithm can improve the robustness of system perception, but when there is no prior information from trusted SUs in the network, this algorithm will not be applicable. In [14], the authors proposed an adaptive clustering defense method for cooperative attack users. It clusters secondary users (SUs) based on the historical sensing information and reputation value of the SUs in the network, reducing the impact of MUs on the global decision results. In [16], the authors considered using the method of double reputation value to maximize the throughput of a CRN network under a small number of SSDF attacks. For SSDF attacks, the optimal trust threshold is derived, but this requires prior information, such as the MU attack probability.

Among the algorithms for defending against SSDF attacks, trust mechanism-based defense algorithms are widely used [14,15,16]. The core idea of the trust mechanism is to construct a trust value for SUs based on their historical sensing information. The trust value of each SU is compared with a pre-set fixed trust threshold. The SUs below the trust threshold will be excluded, or the trust value can be used to assign corresponding weights and penalty values to the sensing results of the SUs. In the trust mechanism, if the fixed trust threshold or weight factor calculation is not accurate enough, MUs cannot be detected, and honest secondary users (HSUs) may be misjudged and assumed to be MUs.

When there are intelligent malicious users (IMUs) in the network, a trust mechanism based on a fixed threshold cannot calculate an appropriate trust threshold due to the lack of prior information, such as the attack probability and proportion of IMUs. This is because, unlike an ordinary SSDF attack, an intelligent malicious user (IMU) who launches an intelligent SSDF attack can dynamically adjust its attack strategies by evaluating its own behavior. During the incubation period, IMUs can upload real results, disguise themselves as honest secondary users (HSUs), and bypass the system’s defense and detection mechanism. For such intelligent SSDF attacks, Feng et al. proposed an additive penalty factor and a multiplicative attenuation factor after analyzing the historical sensing data of malicious users to achieve the dynamic attenuation of their trust values, thereby inhibiting such SSDF attacks [22]. However, such defense methods can easily form false judgments. Once HSUs are marked as MUs, their subsequent sensing results cannot be uploaded to the FC. Zhao et al. introduced a k-means algorithm combined with trust value defense [23] and classified MUs and HSUs through analysis of historical data so as to detect MUs. However, this method only targets a few malicious user scenarios and can only defend against an intelligent SSDF attack with a fixed attack threshold. When the number of IMUs increases and their attack strategies are complex and variable, the detection accuracy of this method is greatly reduced. In [24], Fu et al. proposed the principle of reputation updating based on a cumulative weight sliding window, which assigns less weight to historical sensing observation data, to suppress dynamic attacks. However, when the attack intensity of MUs changes greatly, this method is not applicable.

In order to better defend against the intelligent SSDF attacks described above, this paper introduces the combination of reinforcement learning and a trust value mechanism. Through learning the attack strategies of different MUs, we set corresponding adaptive trust thresholds for different secondary users to ensure the credibility of honest secondary users while identifying malicious users, enabling us to defend against each dynamic attack user and improve the detection performance of the system. The main contributions of this paper are as follows:

- We consider the presence of both ordinary SSDF attacks and more complex intelligent SSDF attacks since IMUs have different attack thresholds and more diverse attack strategies when conducting intelligent SSDF attacks.
- We analyze and summarize the sensing results uploaded by all the secondary users participating in the collaboration and use the trust values established by the beta distribution for each SU to analyze their honesty attributes. We compare the attack intensity of MUs at different periods.
- We introduce reinforcement learning to establish an adaptive trust threshold defense algorithm (ATTR). By analyzing the sensing results of different SUs, we set corresponding trust thresholds for each participating SU to detect malicious users, filter out a set of trusted SUs, and thereby improve the detection probability of the system.

The rest of this article is organized as follows. Section 2 gives the system model and attack model, and Section 3 gives the proposed cooperative spectrum-sensing scheme based on reinforcement learning. Section 4 shows the simulation evaluation of the proposed algorithm and discusses the simulation results. Finally, we give a conclusion in Section 5.

## 2. Preliminaries

### 2.1. System Model

**Figure 1:**
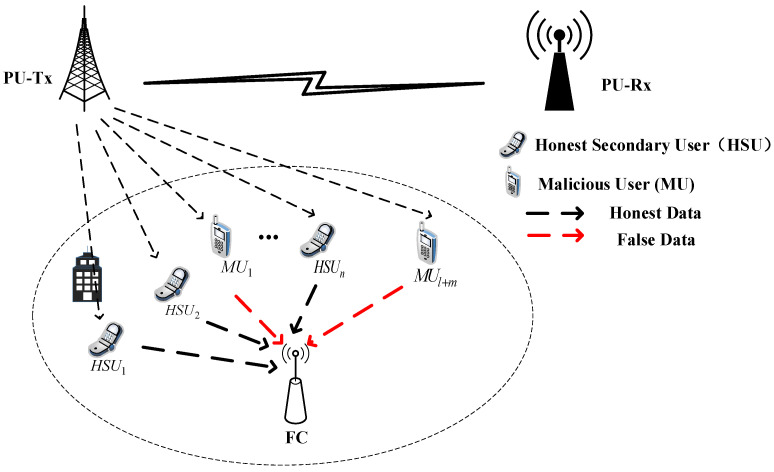
System model frame diagram.

As is shown in Figure 1, in this paper, we consider a centralized cooperative spectrum sensing network with a central base station as the FC, and there is one PU and *N* SUs in the network. The PU communicates on its authorized channel, with PU-Tx as the PU’s signal transmitter and PU-Rx as the PU’s signal receiver. There are two types of secondary users with different honesty attributes among the *N* SUs, namely HSUs and MUs. The HSUs always upload true sensing results in spectrum sensing, while the MUs upload false sensing information based on their attack strategy. The MUs include ordinary MUs and IMUs. The number of HSUs is n, the number of ordinary MUs is l, and the number of IMUs is m. Due to the different locations of the SUs, they are affected by shadow fading and noise, and this results in different detection capabilities.

In the local sensing stage, the spectrum is detected using the energy detection method. Each SU obtains the local sensing result through energy detection and uploads it to the data fusion center (FC). The MU selects an upload result according to its own attack strategy. The energy detection method can describe a binary hypothesis problem:

(1) $$y(t)=\{\begin{array}{ll} n(t), & H_{0}\\ h(t)s(t)+n(t), & H_{1} \end{array},$$

where y(t) is the signal sensed by each SU, s(t) is the transferred PU signal, h(t) is the channel gain on the sensed frequency band, and n(t) is the additive white Gaussian noise on the sensed frequency band. $H_{0}$ and $H_{1}$ represent the hypotheses of the inexistence and the existence of the PU signal.

Firstly, the y(t) signal about the PU received by the SU is sent to the band-pass filter with bandwidth W to filter out the noise signal and obtain the instantaneous energy value $y_{f}(t)$ of the received signal. Secondly, $y_{f}(t)$ is passed through the digital-to-analog converter to obtain the sampling signal of the *N*-point FFT, and then the modulus squared is calculated using the squarer. The calculation result is then integrated within a certain period of time *T* to obtain the test statistic Y. Finally, Y is compared with the preset threshold δ. If Y is greater than δ, the PU is determined to exist in the authorized frequency band; otherwise, the PU does not exist. The detection probability and false alarm probability of local spectrum sensing can be expressed as:

(2) $$P_{d}=P\left(Y>\delta |H_{1}\right)=Q\left(\left(\frac{\delta }{\sigma^{2}}-\gamma -1\right)\sqrt{\frac{N}{4\gamma +2}}\right),$$

(3) $$P_{f}=P\left(Y>\delta |H_{0}\right)=Q\left(\left(\frac{\delta }{\sigma^{2}}-1\right)\sqrt{\frac{N}{2}}\right),$$

(4) $$Q(x)=\int_{x}^{+\infty }\exp\left(-x^{2}/2\right)dx/\sqrt{2\pi },$$

where γ is the signal-to-noise ratio, δ is the energy decision threshold, and $\sigma^{2}$ is the noise variance. After a single sensing, the local sensing result of $SU_{i}$ is obtained, and this is generally represented by binary variables:

(5) $$d_{i}=\left\{ \begin{array}{l} 0,H_{0}\\ 1,H_{1} \end{array}\right..$$

Each SU uploads its local sensing result, and the FC obtains the final global sensing result D, according to the different data fusion rule. Common data fusion methods include the “OR”, “AND”, and “Majority” rules:

- In the “OR” rule, the main idea is that once an SU determines that the channel is occupied, the FC assumes that the PU is using the authorized channel for communication and does not engage in spectrum access. This can protect the normal communication of the PU but reduce the chance of discovering an idle spectrum.
- In the “AND” rule, the FC only considers the PU to be communicating when all the SUs determine that the channel is occupied. This can increase the probability of discovering spectrum voids, but once judged incorrectly it can easily cause interference for the PU.
- In the “Majority” rule, when *K* (K≤N) or more of the *N* SUs participating in the collaboration determine that the channel is occupied, the FC will determine that the PU is communicating.

### 2.2. SSDF Attack Model

In [25], the authors define four types of ordinary SSDF attacks to test the resiliency of the proposed data aggregation scheme:

- “Always yes” attack: regardless of whether the PU exists or not, the MU always uploads to determine the existence of the PU.
- “Always no” attack: regardless of whether the PU exists or not, the MU always uploads and determines that the PU does not exist.
- “Always false” attack: the MU always uploads information that is different from the real sensing result.
- “Fixed probability” attack: the MU uploads false error-sensing information with a fixed probability.

For the above four ordinary SSDF attack models, a large number of trust mechanism-based defense methods have been proposed, but there are few studies on intelligent SSDF attacks. In [23,26], the authors analyze such intelligent SSDF attacks. IMUs will estimate their own trust value $st_{i}$, upload the correct sensing results in the early stage, accumulate their own trust value, and attack when their own trust value reaches $\eta +\lambda_{i}$, where η is the basic trust threshold and $\lambda_{i}$ is the attack threshold of the $IMU_{i}$. At this point, the IMUs have a high trust value and can therefore bypass the traditional trust mechanism.

**Figure 2:**
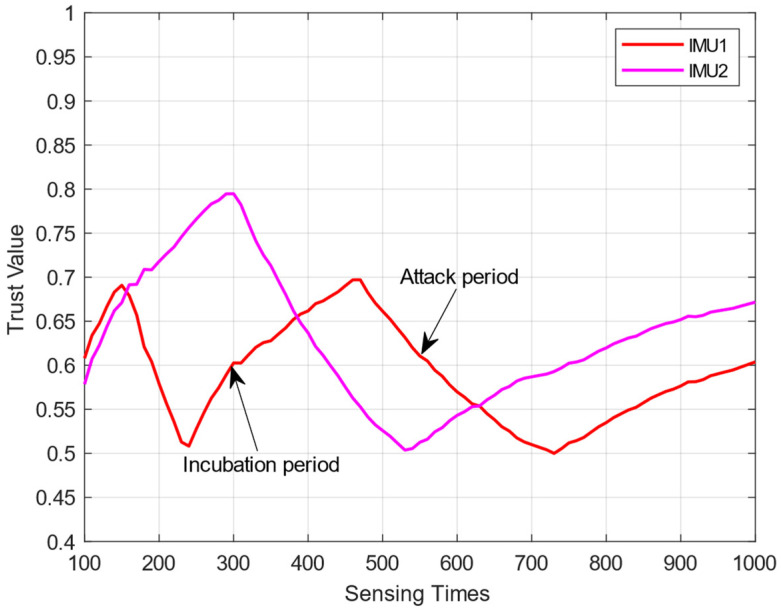
Fluctuation analysis of trust values for IMUs.

In Figure 2, we set two IMUs ($\lambda_{1}=0.2,\lambda_{2}=0.3$). According to the changes in the IMUs’ trust values, they can be divided into an “incubation period” and an “attack period”:

- “Incubation period”: At this time, the trust value $st_{i}$ of the $IMU_{i}$ meets $st_{i}<\eta +\lambda_{i}$. During this period, the $IMU_{i}$ will upload correct sensing results and masquerade as an HSU, thus improving its own trust value so as to hide in the network. The trust value shows an upward trend.
- “Attack period”: At this time, the trust value $st_{i}$ of the $IMU_{i}$ meets $\eta <st_{i}<\eta +\lambda_{i}$. During this period, the $IMU_{i}$ will launch attacks and upload the wrong sensing results. Because the trust value of the $IMU_{i}$ is high at this time, the traditional trust mechanism will consider it an HSU, which affects the detection performance of the system. Due to its upload error-sensing results, its trust value will show a downward trend, and when $st_{i}\leq \eta$, it will enter the “incubation period” again.

In [23], the authors use a k-means algorithm to cluster MUs according to the their simultaneously decreasing trust values. However, it assumes that all the IMUs have the same attack threshold. When the attack threshold is different, this method cannot distinguish HSUs from IMUs, and new methods are needed to prevent such attacks.

### 2.3. Reinforcement Learning in CRN

The core idea of reinforcement learning is to treat agent learning as a Markov decision test process, represented by tuples <S,A,P,R(s,a)>. *S* is the set of environmental states, which indicates that there are a finite number of state sets in the environment. *A* is the set of agent actions, which indicates the finite actions that an agent may send out. *P* is the state transition probability function, which indicates the probability of transitioning from one state to another. *R* (*s*, *a*) is a return function reflecting the learning goal, and it indicates the reward or punishment that can be obtained after an action is issued.

In [27], the authors study the scheduling strategy for different buffers on multiple channels by using Q-learning and deep learning to maximize the system throughput. In [28], the authors formulate the online sequential channel-sensing and -accessing problem as a sequencing multi-armed bandit problem to improve the throughput. For the intelligent malicious attack users proposed above, this paper introduces reinforcement learning to learn their attack strategies.

## 3. Adaptive Trust Threshold Algorithm Based on Reinforcement Learning

By judging the sensing results uploaded by SUs and the global sensing results, we calculate the real-time trust value $st_{i}\in S$ of $SU_{i}$. At the same time, we use the reinforcement learning strategy to iterate the trust threshold of each SU. Finally, we calculate the optimal trust threshold $\eta_{i}$ of $SU_{i}$ so as to obtain the set of HSUs in the cognitive radio network and eliminate the influence of MUs during data fusion.

### 3.1. Trust Value Establishment

By observing the previous perceptual behaviors of cooperative SUs, we found that the two types of perceptual data they send to the FC have binary characteristics: false sensing data and real sensing data. Therefore, the FC can initialize its sensing trust value by using the true sensing times (*TRU*) and false sensing times (*FAL*) of the SUs. The trust values of the SUs that provide real sensing information will be larger, while the trust values of the SUs that continuously provide false sensing information will be smaller. For such binary events, predicting the probability that they will produce favorable events next time is a posterior probability of predicting subsequent behaviors based on historical behaviors [23]:

(6) $$P_{\left(\alpha ,\beta \right)}(x)=P(x|(\alpha ,\beta ))\frac{P((\alpha ,\beta )|x)P(x)}{\sum P((\alpha ,\beta )|x)P(x)}.$$

The probability of binary events can be described using the beta distribution. The known beta probability density function is:

(7) $$\mathrm{Beta}(\alpha ,\beta )=\frac{\Gamma (\alpha +\beta )}{\Gamma (\alpha )\Gamma (\beta )}\theta^{\alpha -1}{\left(1-\theta \right)}^{\beta -1},$$

where θ represents the probability of the occurrence of perceptual behavior, 0≤θ≤1, ∂>0, and β>0, and when ∂<1,θ≠0, 0<β<1,θ≠1. Taking the $SU_{i}$ as an example, $tru_{i}$ and $fal_{i}$ represent, respectively, the number of honest and false perceptions, and thus the formula for calculating the trust value $st_{i}$ can be obtained:

(8) $$st_{i}=\frac{1+tru_{i}}{2+tru_{i}+fal_{i}},st_{i}\in [0,1].$$

### 3.2. Adaptive Trust Threshold Calculation

Q-learning evolves from value iterations in the Markov decision process, but it removes the dependence on transition models, so it is a model-free method. In Q-learning, the agent first selects and performs an action according to the action-selection strategy based on the current state and then calculates the reward according to the reward function. Finally, the agent updates the Q-matrix and jumps to the next state. In the proposed Q-learning framework, the definitions of the state, the action, the reward, the trust threshold, and the trust factor are as follows.

(1) The definition of the state set S represents the trust value set of the SUs and the real-time trust value represented by the state $s=st_{i},s\in S$, which is calculated by comparing the sensing results uploaded by SUs with the global decision results using the aforementioned method of establishing trust values, representing the recognition of each SU by the FC.

(2) The action set is defined as A={0,1} to indicate trust in the SUs. During the update process, the action a∈A should be selected. If a=0, this indicates that the SU is not trusted, and if a=1, this indicates that the SU is trusted.

(3) To define the reward R we compared the upload results of the SUs with the actual channel status and provided feedback on action rewards. When the proportion of MUs is less than 50% of the total number of SUs, the global results of collaborative perception have higher credibility. At the same time, when the FC determines that the channel is idle and allows the SUs to access the channel, action rewards can be evaluated based on whether the access is successful and on the interference feedback when the PU is disturbed.

(4) $\eta_{i}$ represents the real-time trust threshold for $SU_{i}$ and can be compared with the real-time trust value of the SU to determine whether to trust the sensing result of the SU.

(5) κ(0≤κ≤0.1) is the trust factor, which represents the severity of the FC’s decision concerning the maximum trust value of an SU. If its value is too small, it will cause the trust threshold to be set too high, leading to the possible misjudgment of an HSU. Conversely, if its value is too high, it will cause the trust threshold to be set too low, making it impossible to filter MUs.

Policy π: S→A can be defined as follows: In the reinforcement learning process, after each round of spectrum sensing, the data fusion center (FC) calculates the real-time trust value $st_{i}$ of each SU according to the sensing results uploaded by each SU, senses the current state $s_{t}\in S$, and selects the action $a_{t}\in A$ according to the policy π. If it is determined that the SU is trusted, the trust value threshold $\eta_{i}$ of the SU at will be increased. If it is determined that the SU is not trusted, the trust value threshold of the SU will be reduced. According to the trust threshold, the upload results of HSUs are fused according to the K-out-of-N criterion to obtain a new global sensing result *D* and the reward $r_{t}\in R$ of action $a_{t}$. This process is repeated until the optimal strategy is obtained. The status value under policy π is:

(9) $$V^{\pi }(s)=E_{\pi }\left\{r_{t}|s_{t}=s\right\}=E_{\pi }\left\{\sum_{k=1}^{\infty }\beta^{k}r_{t+k+1}|s_{t}=s\right\},$$

where β∈(0,1) represents the discount factor and $V^{\pi }(s)$ represents the expected discount reward. After repeated learning, the optimal action $a^{*}\in A$ is obtained from the maximum cumulative return value over a period of time.

(10) $$Q^{\pi }(s,a)=R(s,a)+\beta \sum_{s\in S}P_{s,s^{\prime }}(a)Q\left(s^{\prime },a\right).$$

The state action value function Q(s,a) is used as the estimation function. After the optimal strategy $\pi^{*}$ is obtained by optimizing the Q-function, the Q(s,a) is updated according to the following formula:

(11) $$Q(s,a)=Q(s,a)+\alpha \left[r_{t}+\gamma \max_{a}Q\left(s^{\prime },a\right)-Q(s,a)\right],$$

where γ is the discount rate and α is the learning rate. The Q-value formula is updated iteratively, and the maximum state action value $\max Q(s^{\prime },a)$ is selected.

By repeating the above process and updating Q(s,a), the optimal trust threshold of each SU is finally obtained. After the adaptive trust threshold $\eta_{i}=\arg\max Q_{i}(s,a=1)-\kappa$ of $SU_{i}$ is obtained, the honest user set Ψ is obtained by comparing the trust value of each SU with its trust threshold. The number of trusted secondary users in set Ψ is defined as ψ:

(12) $$\Psi =\left\{ \begin{array}{l} \Psi \cup SU_{i},\;if\;st_{i}\geq \eta_{i}\\ \Psi ,\;if\;st_{i}<\eta_{i} \end{array}\right..$$

In data fusion, only the sensing result of the trusted secondary user set Ψ is used to eliminate the impact of MUs. The K-out-of-N criterion is used to fuse the data, and the global detection result D is calculated as follows:

(13) $$D=\left\{ \begin{array}{l} H_{0},\;if\;\sum_{i=1}^{\psi }d_{i}<K\;and\;SU_{i}\in \Psi \;\\ H_{1},\;if\;\sum_{i=1}^{\psi }d_{i}\geq K\;and\;SU_{i}\in \Psi \; \end{array}\right.,$$

where $d_{i}$ is the upload local sensing result of $SU_{i}$, K<ψ. The global detection probability $Q_{d}$ and the global false alarm probability $Q_{f}$ of the system are calculated as follows:

(14) $$\left\{ \begin{array}{l} Q_{d}=\sum_{i=K}^{\psi }\left(\begin{array}{l} \psi \\ i \end{array}\right)P_{d}^{i}{\left(1-P_{d}\right)}^{\psi -i},\;SU_{i}\in \Psi \\ Q_{f}=\sum_{i=K}^{\psi }\left(\begin{array}{l} \psi \\ i \end{array}\right)P_{f}^{i}{\left(1-P_{f}\right)}^{\psi -i},\;SU_{i}\in \Psi \end{array}\right..$$

Algorithm 1 shows our proposed ATTR algorithm in detail.
**Algorithm 1:** ATTR Algorithm**Initialization parameters:** Sensing times: *K*; Iteration times of Q-learning: *T*; Learning rate:α; Discount rate:γ. **Input:**   Total SUs: *N*. **Output:**
  The trust threshold of the $SU_{i}$: $\eta_{i}$;  The global detection probability of the algorithm: $Q_{d}$;  The trusted SUs set: Ψ. 1:   **for** *k* = 1 to *K* **do** 2:     **for** *i* = 1 to *N* **do** 3:    $SU_{i}$ perform local spectrum sensing and report the result $d_{i}$ to the FC,      MUs upload according to their own attack strategy; 4:       FC calculates the trust value of the $SU_{i}$,$st_{i}$; 5:       Initialize Q-learning table entry: $Q_{i}(s,a)$; 6:        **for** *t* = 1 to *T*
**do** 7:         Choose action $a_{i,t+1}$ using a policy derived from Q(s,a)i,t;       (e.g., ε-greedy rules) 8:         Update $st_{i}\leftarrow Q_{i}(s,a)$, $\eta_{i,t}\leftarrow \arg\max Q_{i,t}(s,a=1)-\kappa$; 9:      Use the trust threshold $\eta_{i}$ at this time to obtain reward $r_{t}$; 10:         Update $Q_{i,t+1}(s,a)$; 11:      **end for** 12:      Obtain the trust threshold $\eta_{i}$; 13:      **if** $st_{i}>\eta_{i}$ **do** 14:         $\Psi \leftarrow \Psi \cup SU_{i}$; 15:      **end if** 16:    **end for** 17:    Calculate the new global result D via K-out-of-N principle using $SU_{i}\in \Psi$; 18:  **end for** 19:  Calculate the global detection probability $Q_{d}$. 

## 4. Simulation Result

In this section, the simulation results of the proposed algorithm in MATLAB are presented. The general simulation setup is shown in Table 1.

Constant false alarm rate (CFAR) detection is adopted for performance detection, and the global false alarm rate detection probability $P_{f}$ is set to 0.1. In order to verify that our proposed algorithm can effectively detect HSUs and eliminate the influence of MUs during data fusion, we define $Q_{c}$ as the proportion of HSUs correctly identified, which can be calculated as follows:

(15) $$Q_{c}=\frac{\mathrm{Number}\;\mathrm{of}\;\mathrm{HSUs}\;\mathrm{correctly}\;\mathrm{identified}}{\mathrm{Total}\;\mathrm{number}\;\mathrm{of}\;\mathrm{HSUs}}.$$

We also use the final global detection probability to evaluate the performance of the proposed algorithm. To analyze the simulation result of our ATTR algorithm more effectively, we compare it with the TFCA (trust fluctuation clustering analysis) algorithm [23] and SWTM (sliding window trust model) algorithm [24]. The TFCA algorithm is mainly based on the k-means clustering method, which clusters based on decreased trust values when MUs attack simultaneously in order to distinguish between MUs and HSUs. The SWTM algorithm establishes a weighted trust calculation scheme based on multiple small sliding windows using a sigmoid log function to generate the final trust value for each SU, eliminating the need to set the optimal detection threshold. The comparison details are shown in Table 2.

**Figure 3:**
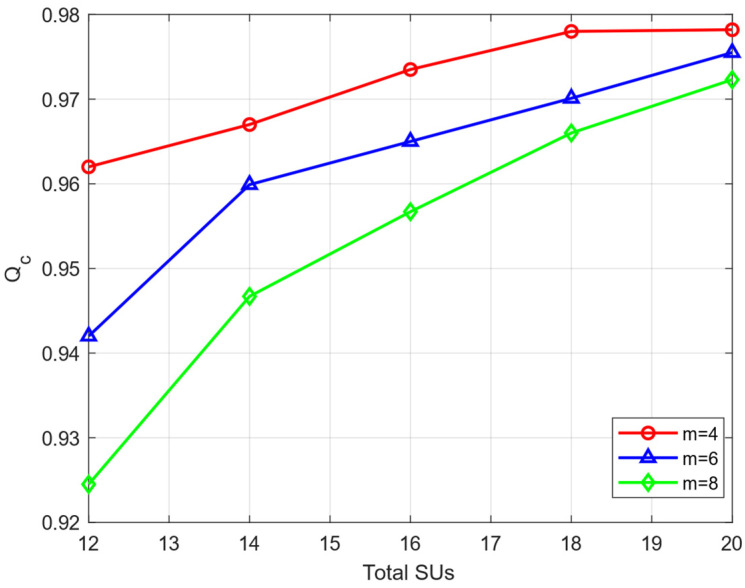
The $Q_{c}$ under different numbers of SUs.

In Figure 3, we compare the changes in $Q_{c}$ value under different circumstances. We set up two ordinary MUs (“always false” attack and “fixed probability” attack) and different numbers of IMUs (*m* = 4, 6, and 8 respectively) with the same attack threshold ($\lambda_{i}=0.2$) who only launch their attacks after their own trust values reach the attack threshold. As can be seen from Figure 3, our proposed algorithm can maintain a high recognition rate for honest users (above 90%). When the number of MUs is small, their impact on the global perception of the system is small, so the HSU recognition rate is higher. When the total number of SUs participating in cooperation increases, the number of MUs remains unchanged, which means that their proportion is reduced, the system is able to detect HSUs more easily, and the HSU recognition rate is improved.

**Figure 4:**
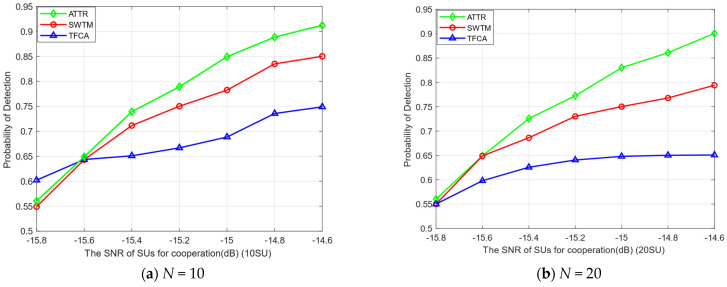
The probability of detection of SNR of SUs.

In the actual cognitive radio network, due to the different environment of each SU, it is affected differently by shadow fading and environmental noise. Therefore, in Figure 4, we compare the advantages and disadvantages of different algorithms in spectrum sensing under different SNR and SU numbers, where IMU accounts for 20% (*λ_i_* = 0.2 and 0.3, respectively). As is shown in Figure 4, with the improvement in the SNR, the SUs’ local detection ability has been improved, and the final global detection performance has also been improved. When the SNR is low, the detection probability of each SU is limited, and the detection probability of each algorithm is low. As the SNR increases, the attack ability of MUs will also increase. Our ATTR algorithm can set a specific trust threshold for each SU according to different situations. At an SNR of −14.6dB, the detection probability of our proposed algorithm can reach over 90%.

**Figure 5:**
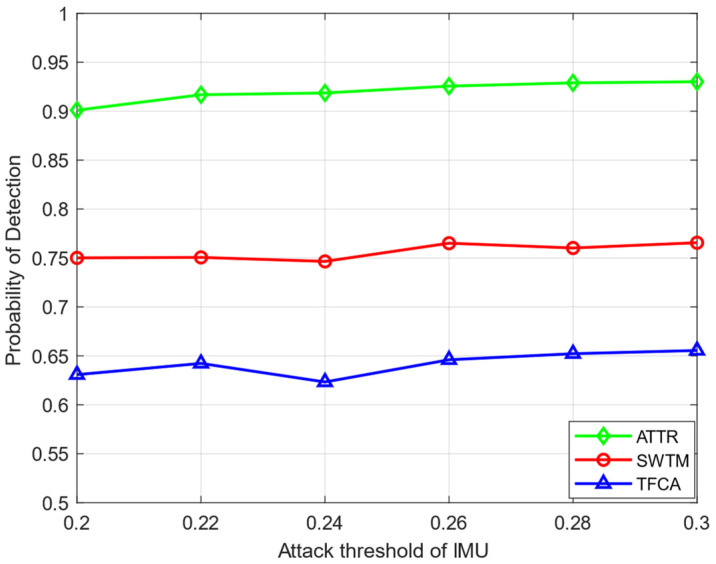
The probability of detection of different attack thresholds.

In Figure 5, we compare the detection performance of each algorithm with the attack intensity of the MUs in the network. In Figure 5, there are 15 HSUs and 5 IMUs. The attack strength, that is, the attack threshold of each IMU, is increased from 0.2 to 0.3. As can be seen from Figure 5, with the increase in the attack threshold of the IMUs, their attack frequency decreases in general, and the overall detection performance improves. The algorithm proposed in this paper has high detection probability and good robustness under different attack thresholds, and the global detection probability can always be maintained at about 92%.

**Figure 6:**
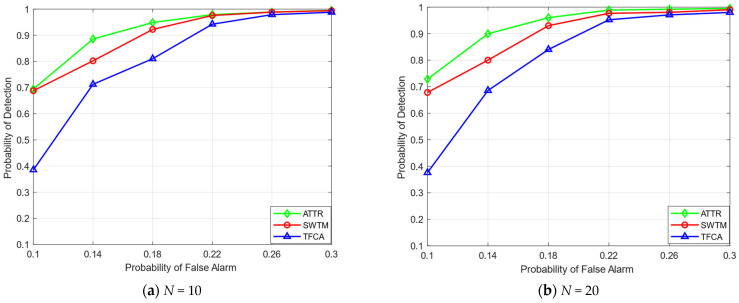
The ROC curves.

In Figure 6, we compare the overall ROC curves, set the false alarm detection probability to between 10% and 30%, set the number of collaborative SUs to 10 in (a) and 20 in (b) (IMU attack threshold $\lambda_{i}\in [0.1,0.3]$), and observe the detection probability of each algorithm. It can be seen from the ROC curves that our proposed algorithm has advantages in overall detection performance. With the improvement of the false alarm detection probability, the detection probability can reach more than 90%.

## 5. Conclusions

This paper introduces the combination of reinforcement learning and a trust mechanism defense. For ordinary SSDF attacks and intelligent SSDF attacks in cooperative spectrum sensing, intelligent SSDF attacks with different attack thresholds are considered, and an adaptive trust threshold defense algorithm based on Q-learning is proposed. By using the trust values established for each SU, using the beta distribution to analyze their honesty attributes, comparing the attack intensity of MUs in different periods, analyzing the sensing results of different SUs, setting corresponding trust thresholds for each participating SU to detect MUs, and filtering out a set of trusted SUs, we improved the detection probability of the system. From the simulation results, it can be seen that the algorithm proposed in this paper can effectively defend against ordinary SSDF attacks and intelligent SSDF attacks in perception networks. In addition, when the proportion of MUs increases and the attack strategy changes, it can also respond well and adjust in time.

## Figures and Tables

**Table 1:** Simulation parameters.

Parameter	Description	Value
*K*	Sensing time	1000
*T*	Iteration time of Q-learning	100
α	Learning rate	0.8
γ	Discount rate	0.8
η	Fixed trust threshold	0.5

**Table 2:** Comparison of different schemes.

Attack Type	TFCA	SWTM	Proposed ATTR
Ordinary SSDF	Medium	High	High
Intelligent SSDF (with same threshold)	High	Medium	High
Intelligent SSDF (with different threshold)	Low	Medium	High

## Data Availability

The data could be obtained from the authors upon reasonable request.
